# It is Not (Always) the Mismatch That Beats You—On the Relationship Between Interaction of Early and Recent Life Stress and Emotion Regulation, an fMRI Study

**DOI:** 10.1007/s10548-021-00880-y

**Published:** 2021-11-14

**Authors:** Andrzej Sokołowski, Monika Folkierska-Żukowska, Katarzyna Jednoróg, Marek Wypych, Wojciech Ł. Dragan

**Affiliations:** 1 Department of Neurology, Memory and Aging Center, UCSF Weill Institute for Neurosciences, University of California, San Francisco, San Francisco, CA USA; 2 Faculty of Psychology, University of Warsaw, Stawki 5/7, 00-183 Warsaw, Poland; 3 Laboratory of Language Neurobiology, Nencki Institute of Experimental Biology of Polish Academy of Sciences, Warsaw, Poland; 4 Laboratory of Brain Imaging, Nencki Institute of Experimental Biology of Polish Academy of Sciences, Warsaw, Poland

**Keywords:** Reappraisal, Emotion regulation, Cumulative stress, Match/mismatch, fMRI

## Abstract

Stress may impact the ability to effectively regulate emotions. To study the impact of stressful experiences in early and recent life on emotion regulation, we examined the relationship between early life stress, recent stress, and brain activation during cognitive reappraisal. We investigated two regulation goals: the decrease and increase of emotional response to both negative and positive stimuli. Furthermore, two models of stress consequences were examined: the cumulative and match/mismatch models. A total of 83 participants (*M*_age_ = 21.66) took part in the study. There was an interaction between cumulative stress and stimuli valence in the cuneus, superior lateral occipital cortex, superior parietal lobule, supramarginal gyrus extending to superior temporal gyrus, and precentral gyrus extending to supplementary motor area. Interaction between mismatched stress index and stimuli valence was found in the left hippocampus, left insula extending to the orbitofrontal cortex and amygdala, and in a cluster including the anterior cingulate cortex, superior frontal gyrus, and frontal pole. Furthermore, there were differences between the effects of cumulative and mismatched stress indices on brain activation during reappraisal of positive but not negative stimuli. Results indicate that cumulative stress and match/mismatch approaches are both useful for explaining brain activation during reappraisal. This finding is important for our understanding of the multifaceted impact of stress on emotion regulation.

**Supplementary Information:**

The online version contains supplementary material available at 10.1007/s10548-021-00880-y.

## Introduction

Emotion regulation is the process of influencing the emotions one has, when one has them, and how one experiences and expresses them (Gross 1998). The process model of emotion regulation is based on cognitive theories of emotions and includes distinct emotion regulation processes (Gross 1998, 2015). One of these processes is cognitive reappraisal, which is a cognitive control process and refers to the reframing of a situation’s meaning in order to change the course of one’s emotions. Reappraisal can be used to decrease as well as to increase emotional response to a situation or stimulus (Gross 2015). Cognitive reappraisal is essential for minimizing the impact of stress; it may determine the degree to which one is resilient or susceptible to stress (Wu et al. 2013). Both acute and past stress may be associated with alterations in emotion regulation processes (Pechtel and Pizzagalli 2011; van Marle et al. 2009). Cognitive reappraisal has been widely studied in neuroimaging studies (for reviews see Morawetz et al. 2017). Reappraisal, as an effortful emotion regulation strategy, mainly engages the prefrontal cortex (PFC) which plays a crucial role in regulatory processes (Dixon et al. 2017)—as per recent meta-analyses, the ventrolateral prefrontal cortex/inferior frontal gyrus (vlPFC/IFG) and dorsolateral prefrontal cortex/middle frontal gyrus (dlPFC/MFG) in particular (Frank et al. 2014; Kohn et al. 2014; Morawetz et al. 2017). These structures receive input from and regulate the activity of the amygdala, which is the central structure which reacts to and is involved in the processing of emotional stimuli (Kohn et al. 2014).

Reappraisal can have one of two goals: downregulation (i.e., decreasing an emotional response and returning to baseline) or upregulation (i.e., increasing the magnitude of an emotional response). While these are both forms of reappraisal, they are two relatively distinct processes and may have partially distinct neural bases (Morawetz et al. 2017). Direct comparison of these goals of regulation shows that decrease of negative emotions is associated with enhanced activation in the right IFG, MFG, and superior frontal gyrus (SFG; Ochsner et al. 2004), while, in contrast, increase of negative emotions involves greater activation in the left SFG, orbitofrontal cortex (OFC), posterior cingulate cortex (PCC), thalamus, and cerebellum. The meta-analysis of Morawetz et al. (2017) examined these different goals separately. Both downregulation and upregulation were found to be associated with activation in the bilateral vlPFC and dlPFC. Direct comparison revealed that downregulation involved greater activation in the right dlPFC and right IPL, while upregulation involved greater activation in the supplementary motor area (SMA) and left insula (Morawetz et al. 2017).

There is a body of literature dealing with the impact of early life stress on emotional regulation. McLaughlin et al. (2015) examined the relationship between the occurrence of physical or sexual abuse in childhood and brain activity during emotion regulation in adolescents. They found that early life stress was associated with increased bilateral activity of the SFG, frontal pole (FP), and anterior cingulate cortex (ACC) during the decrease of emotional response to negative stimuli. These results indicate that adolescents who had experienced early life stress engage the PFC to a greater extent during reappraisal. On the other hand, Kim et al. (2013) showed that childhood poverty was associated with decreased activation in the dlPFC, vlPFC, insula, and temporopolar area as well as enhanced amygdala activation during the downregulation of negative emotional responses. Moreover, functional connectivity analysis indicated that the left amygdala—left vlPFC coupling was positively related to childhood poverty. A longitudinal study by Schweizer et al. (2016) examined the relationship between moderate childhood adversity and brain activation during reappraisal. Individuals who had experienced moderate early life stress showed a greater ability to regulate their negative and positive emotions compared to those who had experienced low stress levels. Participants who had experienced moderate levels of stress exhibited reduced activity in the left amygdala, bilateral MFG, and left middle temporal gyrus (MTG) during negative emotion downregulation as compared to the low stress group. There were no group differences when decreasing positive emotional responses. These results lead to the conclusion that a moderate level of stress in childhood may be beneficial and lead to more effective emotional regulation.

The severity and the timing of stress are both important factors influencing the impact of stress on the brain (McEwen et al. 2015). Childhood is a sensitive time in one’s life as the brain is still in development, and thus early life stress can have serious and lasting consequences, including both cognitive and affective deficits (Pechtel and Pizzagalli 2011). This is why most studies focus on early life stress. However, there is little evidence for persistent effects of stress experienced later in life or the interaction of such stress with early life stress.

To our knowledge, there have been no studies on the impact of the interaction between early and recent life stress on brain functioning during emotion regulation. There are two main approaches used to characterize the consequences of stress: the theory of cumulative stress and the match/mismatch hypothesis (Levine 2005; Nederhof and Schmidt 2012). The first approach relates to the additive effects of stress. One of the potential mechanisms underlying such effects is described by the stress sensitization hypothesis. This says that prior adverse experiences lead to a lower threshold for reactivity to subsequent stressors so that even minor stressors can trigger an emotional response, thus enhancing the risk of psychopathology (Hammen et al. 2000; Szabo et al. 2019). According to this approach, early life stress may negatively affect the development of resilience (Daskalakis et al. 2013). On the other hand, the match/mismatch hypothesis assumes that early life stress promotes the development of coping mechanisms which facilitate effective coping with future adverse events, and thus fosters resilience (Santarelli et al. 2014). According to this model, individuals who experience matched environments (i.e., similar levels of early and recent life stress) should have an advantage in comparison to individuals who experience mismatched environments (i.e., different levels of early and recent life stress; Nederhof and Schmidt 2012). The match/mismatch model has mainly been examined in animal studies, which have suggested that moderate levels of stress in early life can prepare the brain for better functioning under stress (e.g., Oomen et al. 2010). However, recent research on humans has also suggested that the match/mismatch model can shed some light on the mechanisms behind the consequences of stress, linking the match/mismatch model to stress-related psychopathology (Nederhof et al. 2014; Paquola et al. 2017).

Thus, in the current study we examined both the cumulative effects of stress and the effects of mismatched levels of early and recent life stress on cognitive reappraisal in young adults. Note that adults can still be treated as adolescents in developmental terms until the age of 25 (Sawyer et al. 2018). To date, only two human MRI studies have tested both of these approaches simultaneously. Paquola et al. (2017) reported alterations in brain structure and functioning in resting state that were explained by the match/mismatch model, but not the cumulative stress model. However, the cumulative stress model explained the severity of psychopathological symptoms, while the match/mismatch model did not. In our previous study, both cumulative stress and the interaction between early and recent life stress were related to functional connectivity during the processing of facial emotional expressions (Sokołowski et al. 2020), which suggests that the two stress models are not mutually exclusive. So far, no study has looked into these two models in the context of neural activation during cognitive reappraisal. Thus, the aim of this study was to examine brain activation during reappraisal while viewing emotional pictures in non-clinical groups of individuals characterized by different levels of stress experienced in early life and in adulthood. The primary goal of this study was to explore the interaction between life stress, different regulation goals, and stimulus valence on neural activity during cognitive reappraisal of emotional stimuli. The secondary goal was to compare the effects of cumulative life stress and mismatched stress between early and recent life stress on neural activity.

## Methods

### Participants

Out of 90 recruited participants, 83 (41 women) young adults (aged 19–25, M = 21.66; SD = 1.83) took part in the study. One participant was rejected due to MRI contraindication, two did not finish the task, and data from an additional four subjects were discarded due to insufficient coverage of the amygdala in fMRI scanning. Participants were selected from a community sample (*N* = 503) based on Early Life Stress Questionnaire (ELSQ) and Recent Life Changes Questionnaire (RLCQ) outcomes, as measures of early (ES) and recent (RS) life stress. The selection was based on the quartiles of the variables' distributions in the community sample. Random sampling was used to select participants with the lowest and highest scores on both scales. Low levels of stress in childhood and adulthood were operationalized as low scores on the ELSQ and RLCQ, respectively; high levels of stress were operationalized as high scores on these questionnaires. Exclusion criteria were the declared presence of any neurological or psychiatric disorders, traumatic brain injury, addictions to alcohol, drugs, or any other psychoactive substances, as well as any MRI contraindications.

To explore the matched/mismatched model in terms of neural activation, a mismatched stress index was defined as the standardized absolute difference between standardized early and recent life stress scores (where zero indicates a perfect match and the index increases linearly with the level of mismatch). To explore the cumulative stress model, a cumulative stress index was created by adding the standardized scores for the total amount of stress in both life periods.

All participants provided written informed consent and were paid the equivalent of 60 euro in local currency for participating in the study. The procedure was approved by the Ethics Committee at the University of Warsaw. The study was conducted in accordance with the guidelines of the Declaration of Helsinki.

### Assessment

The Early Life Stress Questionnaire (ELSQ; Cohen et al. 2006; Sokołowski and Dragan 2017) was used to assess early life stress. It measures exposure to 19 stressful events such as emotional, sexual, physical abuse, violence, negligence, parental divorce, surgery, parental death, separation, etc. Participants indicate whether they experienced any of these events before the age of 12, with a maximum score of 19. Reliability was assessed by estimating internal consistency using Cronbach’s α. Cronbach’s α in our sample was .85.

The Recent Life Changes Questionnaire (RLCQ; Rahe 1975; Sobolewski et al. 1999) was used to measure the level of stress in adulthood. It covers events such as the death of a loved one, divorce, injury, loss of income, etc. Participants were asked whether they had experienced any such stressful events in the previous 24 months, with a maximum score of 73. Cronbach’s α in our sample was .94.

### Experimental Task

Three fMRI tasks were undertaken as part of a larger project, and the data from the reappraisal task is presented here. A set of 112 color emotional images was used in the task: 48 positive, 48 negative, and 16 neutral pictures (see Supplementary Material). The stimuli were taken from the Nencki Affective Picture System (NAPS; Marchewka et al. 2014). Pictures showed social situations, people, and faces. Stimuli were presented against a grey background using the Presentation software (Neurobehavioral Systems, Inc., Berkeley, CA). Instructions and training regarding regulation strategies were given prior to the scan. Participants were familiarized with the scanner and the experimental procedure in a mock scanner. Participants verbalized their understanding of the task to a researcher and practiced the strategies using different stimuli. Feedback was given until they established effective strategy usage.

The cognitive reappraisal task was adapted from previous studies (Kim et al. 2013; McLaughlin et al. 2015; Ochsner et al. 2004). Two emotion regulation goals (decrease and increase of emotions) and a control condition (passive viewing) were used in a block design. In the decreasing condition, participants were instructed to downregulate their emotional response by reinterpreting the situation in a less negative/positive way, rationalizing (perceiving the situation in a more objective way), or self-distancing (becoming a detached observer, making the situation psychologically distant). In the increasing condition, participants were instructed to upregulate their emotional response by reinterpreting the situation in a more negative/positive way, engaging with the situation, increasing subjective closeness, or perceiving the situation as real. The regulation instructions were applied only to emotional conditions (positive and negative). In the look condition (applied to emotional and neutral stimuli), participants were asked not to modulate their emotional response and to look at the presented pictures without engaging any regulation strategy. Participants were instructed to use the strategies that are most suitable, they feel the most familiar with, and would be the most effective in the particular situation. The strategies the participants actually used were not controlled. Affect rating was registered as a behavioural measure after each block using a visual scale. See Supplementary Material for task design details.

### Behavioural Data Analysis

In order to examine whether affect rating differed between regulation goals, repeated measures ANOVAs were performed. To test whether cumulative stress or mismatched stress predicted affect rating, a regression analysis was performed with stress as predictor and affect rating as dependent variable.

### MRI Data Acquisition and Preprocessing

Whole–brain functional and structural images were acquired using a 3T MRI scanner (Trio TIM, Siemens, Germany) equipped with a 32-channel head coil. First, a localizer and high-resolution T1-weighted images were obtained with the following parameters: TR/TI/TE = 2530/1100/3.32 ms; flip angle = 7°; PAT factor = 2; FoV = 256 mm; voxel dimensions = 1 mm isotropic; 256 × 256 voxel resolution. Functional images were acquired using a T2-weighted, gradient-echo echo planar imaging (EPI) pulse sequence during a single functional run. A total of 570 whole–brain volumes were recorded with the following parameters: TR/TE = 2000/30 ms; flip angle = 90°; 64 × 64 matrix size; FoV = 224 mm; 3.5 × 3.5 mm vox size; 35 slices (interleaved ascending); 3.5 mm slice thickness.

Preprocessing was performed using Statistical Parametric Mapping (SPM12, Wellcome Department of Cognitive Neurology, London, UK), implemented in MATLAB (2018, The Math-Works Inc. Narick, MA, USA). Field map scans were used to minimize geometrical distortions in images caused by field inhomogeneities. During preprocessing, functional images were spatially realigned, slice-time corrected (to the middle slice), coregistered to the first functional image, normalized to the standard MNI template (based on anatomical image), and spatially smoothed with an 8 mm isotropic Gaussian kernel.

### First-Level Analysis

Data from a single experimental run were modeled with a general linear model (GLM) for each participant using SPM12. The following regressors of interest were added to the model (as blocks in a block design): (a) decrease negative; (b) decrease positive; (c) increase negative; (d) increase positive; (e) look negative; (f) look positive; and (g) look neutral. The following regressors of no interest were put into the model: (h) instructions; (i) affect rating, and (j) fixation crosses. Each regressor was convolved with a canonical hemodynamic response function. The model also included an additional 6 regressors of no interest to account for head motion. The Artifact Detection Tool (ART) toolbox was used to determine extended head movements, and volumes exceeding 2 mm or .05 rad movement thresholds were regressed-out from analyses (mean number of excluded volumes was .169).

### Second-Level Analysis

Single-participant contrasts for the four reappraisal task conditions were submitted to second-level analyses in SPM12: (1) decrease negative > look negative; (2) increase negative > look negative; (3) decrease positive > look positive; and (4) increase positive > look positive. To test the match/mismatch hypothesis, the mismatched stress index was used (as an absolute difference between early and recent life stress). To test the cumulative stress hypothesis, the cumulative stress index was used as the sum of standardized early and recent life stress scores. A 2 × 2 flexible factorial model was used with stimuli valence (negative, positive) and regulation goals (increase, decrease) and their interactions with the two stress indices. To compare the cumulative and mismatch models, the two stress regressors were directly compared for each of four reappraisal task conditions. Family-wise error (FWE) correction was used to control for multiple comparisons in whole–brain analysis (*p* < 0.001 height-threshold; FWE < .05 extent-threshold).

## Results

### Behavioural Results

Across all participants, a repeated measures ANOVA determined that affect rating differed between task conditions (*F*_2, 164_ = 77.88; *p* < .001; η_p_^2^ = .49). Post-hoc tests with Bonferroni correction revealed higher affect ratings in the increasing condition (*M* = 40.51; *SD* = 17.03) compared to the look condition (*M* = 31.64; *SD* = 14.50; *p* < .001), while ratings in the decrease condition (*M* = 23.57; *SD* = 11.51) were lower than in the look condition (*p* < .001).

In order to examine the effects of stress on affect rating, regression analysis was performed. Cumulative stress and mismatched stress did not predict affect ratings across conditions (all *p*’s > .05).

### Interaction Between Stress and Stimuli Valence

Across all participants, whole–brain analysis of the main effect of task (decrease + increase > look) revealed robust activation mainly in the bilateral prefrontal cortex (vlPFC, dlPFC, SFG, OFC, insula), temporal lobe (MTG, temporal pole, TP), and bilateral subcortical structures (thalamus, putamen, caudate). There was deactivation in the medial cortex (SMA, PCC), parieto-occipital cortex (precuneus, cuneus), and somatomotor cortex (see Supplementary Table S2 and Fig. S2).

**Fig. 1:**
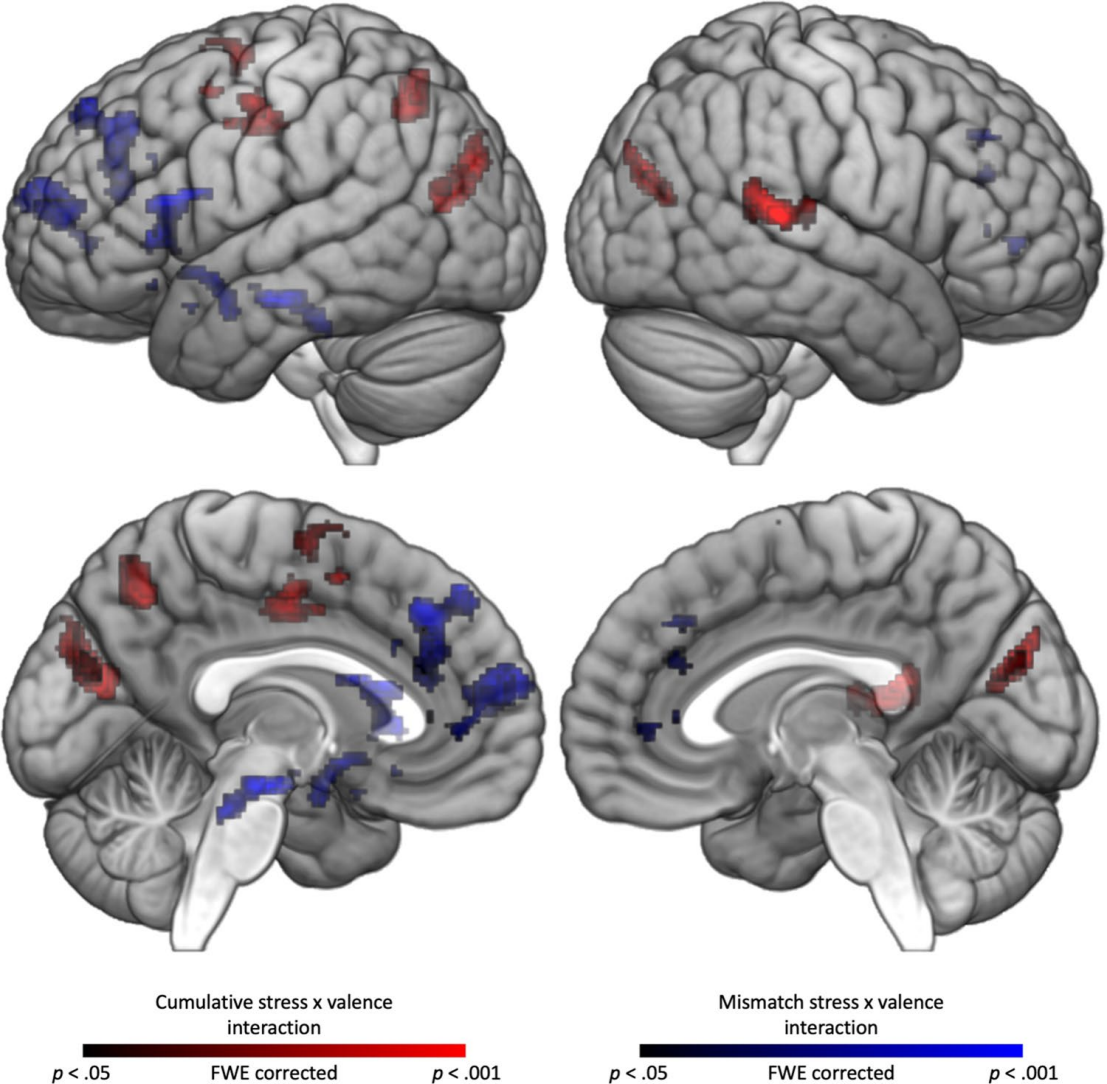
Interaction effects between stress and stimulus valence on brain activation. Regions with significant interaction between cumulative stress and valence are shown in warm colors, regions with significant interaction between mismatched stress and valence are shown in cold colors. *sLOC* superior lateral occipital cortex, *SMG* supramarginal gyrus, *SPL* superior parietal lobule, *STG* superior temporal gyrus

**Fig. 2:**
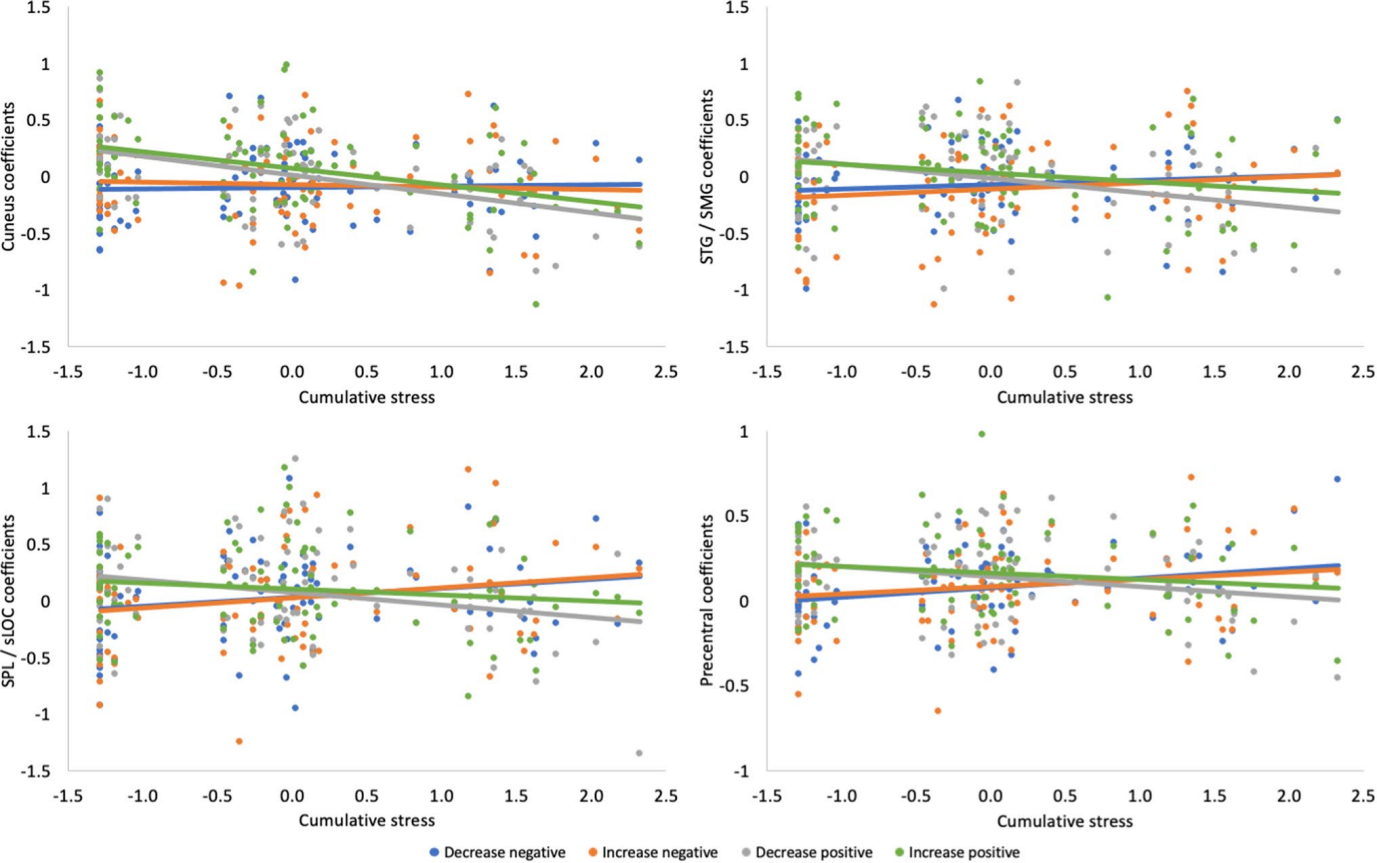
Interaction effects between cumulative stress and stimulus valence on brain activation. Regression slopes for all task conditions are presented. Standardized cumulative stress scores are shown on the x axis. *sLOC* superior lateral occipital cortex, *SMG* supramarginal gyrus, *SPL* superior parietal lobule, *STG* superior temporal gyrus

There was a significant interaction between cumulative stress and stimulus valence in four brain regions: cuneus, SMG extending to STG, sLOC and SPL, and precentral gyrus extending to SMA. For participants with higher cumulative stress, activation in these regions was higher during reappraisal of negative stimuli and lower during reappraisal of positive stimuli. Inversely, activation was higher during reappraisal of positive stimuli and lower during reappraisal of negative stimuli in those with lower cumulative stress. The results are reported in Table 1 and illustrated in Figs. 1 and 2.

**Table 1:** Interaction between stress and stimulus valence in whole–brain activation during reappraisal

Contrast and brain region(s)	Cluster Size (voxels)	*p* value for cluster (FWE)	*F*	x	y	z
*Cumulative stress × valence*						
Cuneus	1223	< .001	25.36	10	− 78	24
SMG/STG	431	.011	25.13	62	− 38	14
SPL/sLOC	314	.037	22.19	− 20	− 62	48
Precentral gyrus/SMA	622	.002	20.61	− 32	− 12	42
*Mismatched stress × valence*						
Hippocampus	303	.042	26.84	− 16	− 16	− 18
Insula/OFC/Amyg	804	< .001	26.1	− 28	18	16
SFG/ACC/FP	1757	< .001	21.55	− 12	58	16

*ACC* anterior cingulate cortex, *Amyg* amygdala, *FP* frontal pole, *OFC* orbitofrontal cortex, *SFG* superior frontal gyrus, *sLOC* superior lateral occipital cortex, *SMA* supplementary motor area, *SMG* supramarginal gyrus, *SPL* superior parietal lobule, *STG* superior temporal gyrus

**Fig. 3:**
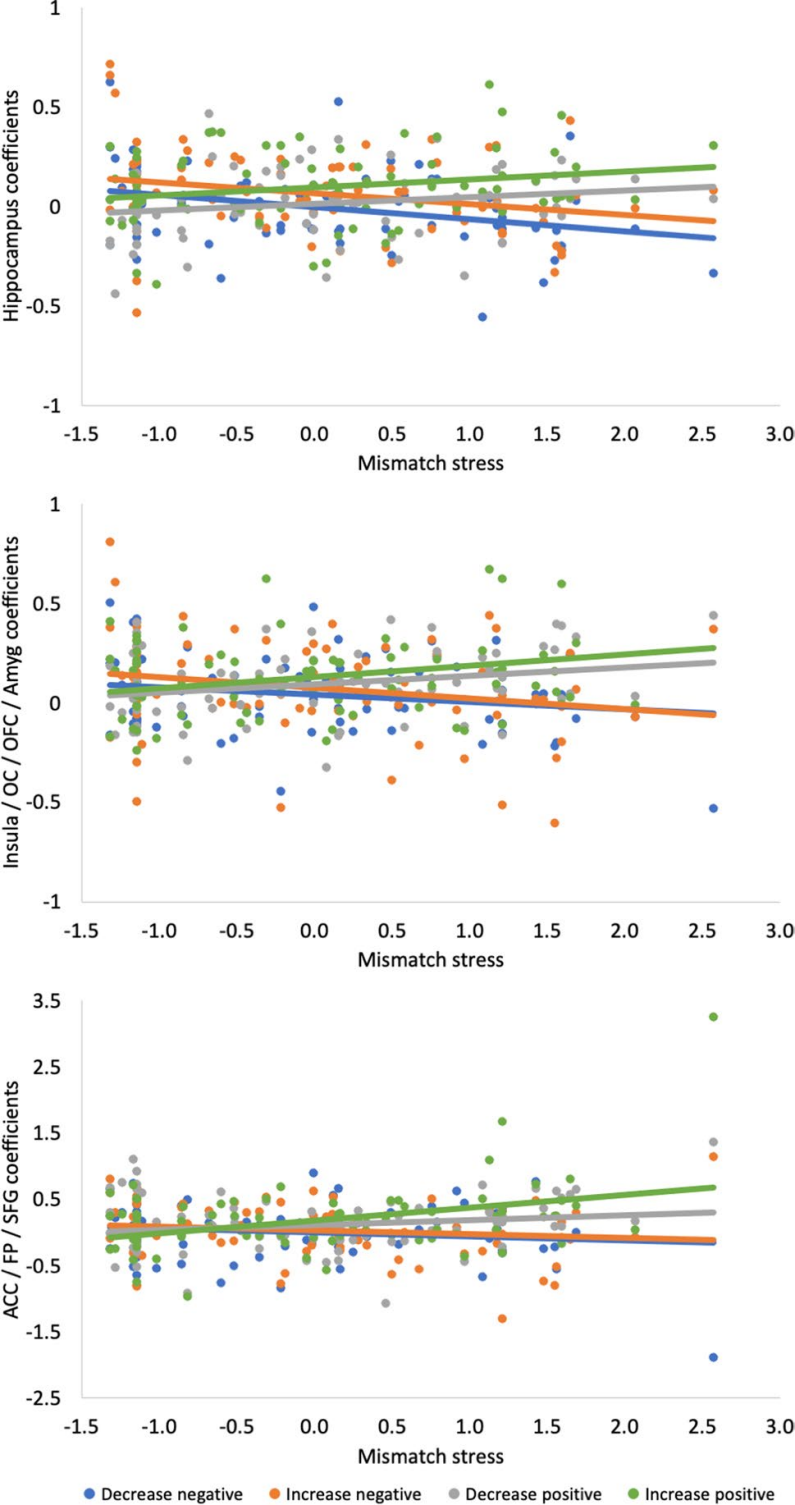
Interaction effect between mismatched stress and stimulus valence on brain activation. Regression slopes for all task conditions are presented. Standardized mismatched stress scores are shown on the x axis. *ACC* anterior cingulate cortex, *Amyg* amygdala, *FP* frontal pole, *MFG* middle frontal gyrus, *OC* opercular cortex, *OFC* orbitofrontal cortex

Analysis of the interaction between mismatched stress and stimulus valence yielded significant results in three brain areas. The first cluster involved the left hippocampus, the second cluster involved the left insula extending to the OFC and amygdala, and the last cluster included ACC, SFG, and frontal pole. In contrast to the effect of cumulative stress, higher levels of mismatched stress were positively related to brain activation during reappraisal of positive emotions and negatively during reappraisal of negative emotions. Activation in those participants with low levels of mismatched stress (thus, levels of early and recent life stress were matched) was somewhat similar regardless of the stimulus valence. Results are reported in Table 1 and illustrated in Figs. 1 and 3.

There was no significant interaction between cumulative stress or mismatched stress and regulation goal related to whole–brain activation (*p* > .05). Additional analyses for the effects of early and recent life stress are reported in the Supplementary Material.

### Effects of Cumulative and Mismatched Stress on Whole–Brain Activation in the Four Task Conditions

**Fig. 4:**
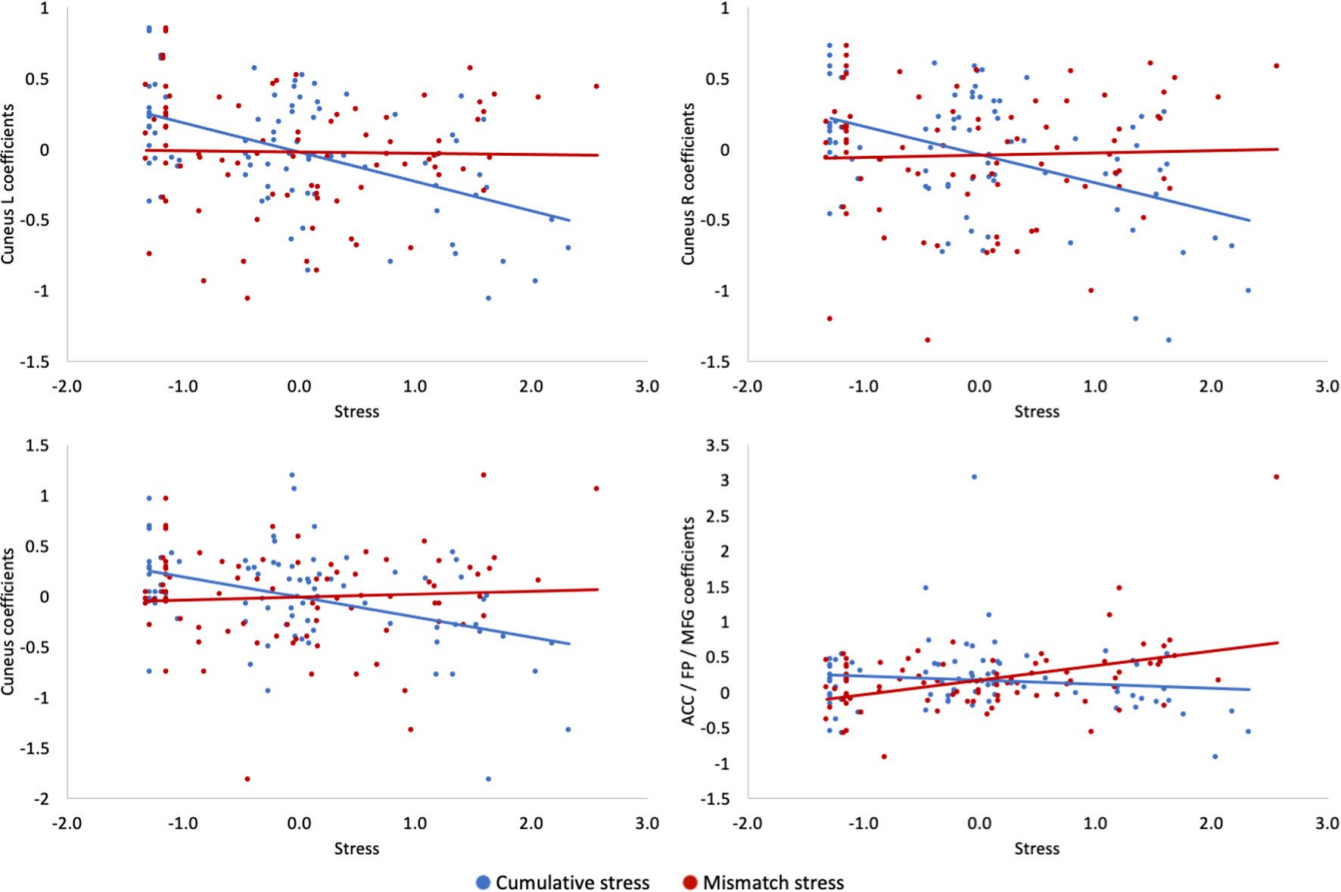
Differences between the effects of cumulative and mismatched stress on brain activation during decreasing (top panels) and increasing (bottom panels) positive emotions. Standardized stress scores for cumulative and mismatched stress are presented on the x axis. *ACC* anterior cingulate cortex, *FP* frontal pole, *MFG* middle frontal gyrus

There was a significant difference between the effects of cumulative and mismatched stress on brain activation during increasing and decreasing positive emotions (Tables 2, 3, Fig. 4). The cuneus showed stronger positive relation to mismatched stress than cumulative stress during the processing of positive stimuli regardless of the regulation goal. This difference was driven by a significant negative relationship between cumulative stress and cuneus activation. The effect of mismatched stress was also stronger than cumulative stress in a region consisting of ACC and MFG. This difference was driven by a significant positive relationship between mismatched stress and activation in these regions. There was no effect of cumulative or mismatched stress on reappraisal-related brain activation for the decrease negative > look negative and the increase negative > look negative conditions.

**Table 2:** Reappraisal-related brain activation for the decrease positive > look positive contrast

Contrast and brain region(s)	Cluster size (voxels)	*p* value for cluster (FWE)	*t*	x	y	z
*Negative correlation with cumulative stress*						
Cuneus	2137	0	5.47	− 16	− 80	24
*Mismatched* > *cumulative stress*						
Cuneus	414	0.007	4.31	14	− 80	32
Cuneus	334	0.018	4.18	− 16	− 72	22

**Table 3:** Reappraisal-related brain activation for the increase positive > look positive contrast

Contrast and brain region(s)	Cluster size (voxels)	*p* value for cluster (FWE)	*t*	x	y	z
*Negative correlation with cumulative stress*						
Cuneus	1456	0	5.06	12	− 76	24
*Positive correlation with mismatched stress*						
ACC/FP/MFG	2279	0	5.36	− 26	54	10
MFG/FP	529	0.004	4.18	46	32	30
*Mismatched* > *cumulative stress*						
Cuneus	840	0	4.64	20	− 72	24
ACC/FP/MFG	1598	0	4.58	− 26	52	10

## Discussion

The present study examined the relationship between stress accumulated during one’s lifetime and brain activity during cognitive reappraisal using two models of stress consequences: one based on the match/mismatch hypothesis and the other based on the cumulative stress hypothesis. The first model examined the effect of mismatch between stress in early and recent life. The second model investigated the additive effect of stress experienced over both periods. The main aim of this study was to examine the interaction between stress and different regulation goals as well as stimulus valence during cognitive reappraisal. We also compared the effects of cumulative and mismatched stress on neural activity.

To our knowledge, no previous attempt has been made to investigate the cumulative and match/mismatch models of stress exposure in the context of cognitive reappraisal. It is also worth noting that our study included two reappraisal goals—upregulation and downregulation—as well as both positive and negative valence stimuli. Many previous studies only used one type of reappraisal and one type of stimulus.

There was an interaction between cumulative stress and stimulus valence in brain activation during reappraisal. Activation in regions including the bilateral cuneus, right SMG, left SPL, and left precentral gyrus were different during reappraisal of positive and negative stimuli depending on the level of cumulative stress. The cumulative effect was strongest for the cuneus, where the main effect of cumulative stress was also significant. The cuneus has previously been shown to be involved in cognitive reappraisal (Goldin et al. 2008) and the processing of emotional expressions during mimicking and suppressing one’s own reactions (Vrticka et al. 2013). SPL has also been described as a region supporting reappraisal (Buhle et al. 2014). The activation observed in the cuneus and SPL could be partially driven by the effect of early life stress. The activity of the occipital and parietal cortices may be related to both the processing of complex visual stimuli (here photos depicting social situations) and the use of emotion regulation strategies. This might suggest that cumulative stress impacts the processing of the valence aspect of emotional social stimuli. The SMG is a structure engaged in directing attention to the emotional aspects of a stimulus (Ochsner et al. 2012) and the processing of emotionally salient stimuli (Santos et al. 2011). Loeffler et al. (2019) studied a sample of healthy participants and patients with depression and reported that the SMG was involved in the attentional control of emotions and cognitive reappraisal. Interestingly, the common activation was significant for positive but not negative emotions. It suggests that cumulative stress in our sample could have altered the processing of positive stimuli and could potentially lead to decreased attention to positive stimuli. Wadden et al. (2018) indicated that individuals who practice yoga or meditation (known to decrease stress and increase attention) have increased SMG and SPL reactivity to emotional stimuli. The higher cumulative stress in our sample may be related to decreased attention to positive stimuli. It is unclear whether the altered activation of the precentral gyrus and SMA is related to cumulative stress, but it may be related to less self-involvement in positive stimuli (Frank et al. 2014). SMA has been consistently shown to be activated during the reappraisal process, both increasing and decreasing emotions, independently of the emotion regulation strategies used (Morawetz et al. 2017).

The dependence of the effect of cumulative stress on brain activation on stimulus valence can be interpreted in the context of reward processing. Morelli et al. (2021) reported that children with higher levels of early life stress (controlled for the presence of recent stress) display decreased brain activation in response to reward anticipation and increased activation when a reward is not expected. The authors suggest that adverse events early in life may lead to altered neuronal response in individuals who experienced an unpredictable environment and lack of rewards. Hence, the difference in activation while processing positive and negative stimuli in our study may be related to a lower recurrence of rewards and more frequent punishments in participants with higher levels of cumulative stress.

The interaction between mismatched stress and stimulus valence revealed that a greater mismatch between early and recent stress is related to deactivation during reappraisal of negative stimuli but activation during reappraisal of positive stimuli in various brain structures. This effect was present in subcortical structures (the hippocampus and amygdala). The amygdala is a structure highly involved in emotional reactivity while the hippocampus provides episodic context related to previous experience (Etkin et al. 2015). Their activation depended on the mismatched stress interaction with stimulus valence but not the regulation goal, being more engaged in processing positive stimuli in participants with higher mismatched stress. This may suggest that participants with higher mismatched stress indices are more emotionally reactive to positive than negative stimuli and perceive positive stimuli as being more emotionally salient. This stands in contrast to the notion that the amygdala is more reactive to negative stimuli (such as threats) than positive ones (Ochsner et al. 2012). The amygdala is not only involved in salience detection but also takes part in shifting from effortful cognitive regulation to more rigid habitual coping (which may be less effective) in response to stress (Schwabe and Wolf 2013). This is in-line with the assumption underlying the match/mismatch hypothesis that exposure to mismatched stress is less adaptive (Nederhof and Schmidt 2012).

Furthermore, it has been previously shown that increased activity of the amygdala is related to self-consciousness during emotional suppression (Chen et al. 2017). The hippocampus provides episodic context to self-referential memories and its engagement in memory retrieval depends on self-involvement (Muscatell et al. 2010). Self-consciousness, along with vulnerability to stress, is considered to be one of the subtraits of neuroticism (John et al. 2008). Self-consciousness is associated with the processing of social emotions and emotion regulation as well as being susceptible to stress (Haga et al. 2009; Takishima-Lacasa et al. 2014). Haga et al. (2009) indicated that self-consciousness is related to the habitual use of cognitive reappraisal. Interestingly, both non-adaptive neuroticism in the study of Chen et al. (2017) and unfavourable stress (i.e., mismatched stress exposure) during the regulation of positive emotions in our study were related to increased amygdala activity. Thus, we propose that activity of the amygdala and hippocampus is not solely associated with affective suppression but also with the cognitive reappraisal of emotions, potentially undergirding a more general mechanism of emotion regulation processes that is sensitive to stress exposure.

An interaction effect was present in the anterior insula and ACC. These structures are part of the salience network which is responsible for the identification of salient information and directing attention to affective stimuli (Uddin et al. 2019; Yeo et al. 2011). Our results suggest that this network may be more engaged in the processing of positive stimuli than negative stimuli in individuals with mismatched stress experience. They can either perceive positive stimuli as more relevant or avoid focusing on negative stimuli. This would suggest the presence of positivity bias when positive stimuli are perceived as more salient, especially when this is in-line with the current evaluative goal (Cunningham et al. 2008; Mather et al. 2004). Mismatched stress may impact attentional deployment even before effortful processes such as cognitive reappraisal are implemented. Affect-based attention, defined as a predisposition towards focusing on particular features of emotional stimuli, is a relevant aspect of emotion regulation (Todd et al. 2012). Insufficient attention to negative stimuli in individuals with mismatched stress could result in inefficient emotion regulation.

An alternative interpretation of the results is that individuals who experienced mismatched stress have an advantage. They may prioritize negative stimuli relevant in social contexts based on their history of adverse experiences, which would explain the diminished amygdala activation during reappraisal of negative stimuli, as is expected during effective emotion regulation. Amygdala activation during reappraisal is a correlate of successful emotion regulation (Wager et al. 2008). If this interpretation were accurate, it could mean that the mismatch hypothesis should be modified to take into account that individuals who experienced mismatched stress are, in fact, more effective at regulating negative than positive stimuli, while individuals with matched stress regulate positive and negative stimuli in the same way.

We investigated the difference between how cumulative and mismatched stress are related to brain activation during reappraisal. The effects of cumulative stress and mismatched stress were different in the cuneus as well as the ACC and MFG during the processing of positive stimuli. The differences were driven by significant main effects of stress. Cuneus activation was negatively related to cumulative stress regardless of the regulation goal. Corticosteroids influence sustained attention by decreasing cuneus activity which, in turn, may lead to altered stimulus-driven attentional processing (Hahn and Stein 2006; Henckens et al. 2012). The cuneus is engaged in emotional attention to positive but not negative facial expressions (although this relationship decreases with age; Lindstrom et al. 2009). Given that socioemotional images in our study often depicted faces, it’s possible that cumulative stress may have impacted the attention to positive stimuli. The cuneus is also engaged in aesthetic appreciation, which may explain the presence of increased aesthetic preference during the processing of positive images (Mizokami et al. 2014; Zhang et al. 2017).

The ACC and MFG are both highly involved in cognitive reappraisal. The prefrontal cortex is crucial for top-down processes and cognitive control over emotional arousal (Kohn et al. 2014). The ACC has been found to be more involved in the processing of positive than negative stimuli (Vrtička et al. 2011). It also takes part in integrating cognitive and emotional processes (Bush et al. 2000). Greater involvement of these structures in individuals with mismatched stress can be seen as a compensation mechanism that may attenuate potential deficits that could be a result of mismatched stress.

It is important to note that stress-related alterations in brain activity when performing tasks related to emotion regulation processes can be explained in two ways: as a sign of a deficit or of compensation. The first explanation assumes a lesser ability to effectively implement these processes, reflected in the weaker or ineffective involvement of brain structures (Etkin et al. 2010). The second possible mechanism assumes the need for engagement of more cognitive and neural resources to effectively regulate emotions (Etkin and Schatzberg 2011).

Our results differ from those obtained by Paquola et al. (2017), who only found a match/mismatch effect and no impact of cumulative stress on resting state functional connectivity. The implementation of a task-based approach in our study allowed the detection of effects related to both the cumulative stress and match/mismatch models. The notion that both approaches are credible is in line with our previous study that showed the effect of both cumulative stress and an interaction between early and recent life stress on the processing of emotionally salient stimuli, namely emotional facial expressions (Sokołowski et al. 2020). Moreover, the effects were independent of regulation goals.

Our study has certain limitations. The first is the use of an additive index to measure early life and recent stress, without differentiation between the types of stressors—it is known that distinct adverse events may have at least partially different consequences (McLaughlin and Sheridan 2016). While stressful events can be characterized as differing in the extent of deprivation and threat, it is also possible to differentiate them on the basis of situations directly concerning oneself and those affecting people in one’s environment, and thus having an indirect impact (Dragan 2018; Palgi et al. 2012), or to differentiate between brief (acute) and chronic stressors (Franklin et al. 2012). Combined measures of stressful events, although commonly used in research, make it difficult to analyse specific consequences of particular adverse events. While participants were encouraged to implement various cognitive strategies to regulate their emotions, we did not control what strategies were actually used during task performance within the scanner. It has been previously shown that different regulation strategies may involve different neural activation (Goldin et al. 2019; Ochsner and Gross 2005). Lastly, since individuals can be considered as adolescents until the age of 25 (Sawyer et al. 2018), our results should not be generalized to the entire adult population. In this context, it is worth noting that McRae et al. (2012) showed that involvement of cognitive control during reappraisal increases linearly with age.

This study has demonstrated that models based on both the cumulative stress and the match/mismatch hypotheses can be useful in describing the effects of early life stress and recent stress in adulthood on the brain, including its functioning during cognitive reappraisal. Our results showed that stress affected brain activation observed during emotion reappraisal in areas linked to emotion processing. This could be an indication of specific effects of stress levels in early life and in adulthood on general mechanisms of emotion regulation. The effects of cumulative stress and mismatched stress depended on stimuli valence, suggesting that stress has a different impact on brain activation during regulation of positive and negative emotions. Future studies are needed to explore the differences in the effects of early and recent stress as well as their interaction with emotional processing.

## Supplementary Information

Below is the link to the electronic supplementary material.

Supplementary file1 (PDF 1491 kb)

## Data Availability

The data that support the findings of this study are available from the corresponding author upon reasonable request.

## Code Availability

The data that support the findings of this study are available from the corresponding author upon reasonable request.

## References

[CR1] Buhle JT, Silvers JA, Wager TD, Lopez R, Onyemekwu C, Kober H, Ochsner KN (2014). Cognitive reappraisal of emotion: a meta-analysis of human neuroimaging studies. Cereb Cortex.

[CR2] Bush G, Luu P, Posner MI (2000). Cognitive and emotional influences in anterior cingulate cortex. Trends Cogn Sci.

[CR3] Chen S, Chen C, Yang J, Yuan J (2017). Trait self-consciousness predicts amygdala activation and its functional brain connectivity during emotional suppression: an fMRI analysis. Sci Rep.

[CR4] Cohen RA, Hitsman BL, Paul RH, McCaffery J, Stroud L, Sweet L, Gunstad J, Niaura R, MacFarlane A, Bryant RA, Gordon E (2006). Early life stress and adult emotional experience: an international perspective. Int J Psychiat Med.

[CR5] Cunningham WA, Van Bavel JJ, Johnsen IR (2008). Affective flexibility: evaluative processing goals shape amygdala activity. Psychol Sci.

[CR6] Daskalakis NP, Bagot RC, Parker KJ, Vinkers CH, de Kloet ER (2013). The three-hit concept of vulnerability and resilience: toward understanding adaptation to early-life adversity outcome. Psychoneuroendocrinology.

[CR8] Dixon ML, Thiruchselvam R, Todd R, Christoff K (2017). Emotion and the prefrontal cortex: an integrative review. Psychol Bull.

[CR9] Dragan M (2018). Adverse experiences, emotional regulation difficulties and psychopathology in a sample of young women: model of associations and results of cluster and discriminant function analysis. Eur J Trauma Dissoc.

[CR10] Etkin A, Schatzberg AF (2011). Common abnormalities and disorder-specific compensation during implicit regulation of emotional processing in generalized anxiety and major depressive disorders. Am J Psychiatry.

[CR11] Etkin A, Prater KE, Hoeft F, Menon V, Schatzberg AF (2010). Failure of anterior cingulate activation and connectivity with the amygdala during implicit regulation of emotional processing in generalized anxiety disorder. Am J Psychiatry.

[CR12] Etkin A, Büchel C, Gross JJ (2015). The neural bases of emotion regulation. Nat Rev Neurosci.

[CR13] Frank DW, Dewitt M, Hudgens-Haney M, Schaeffer DJ, Ball BH, Schwarz NF, Hussein AA, Smart LM, Sabatinelli D (2014). Emotion regulation: quantitative meta-analysis of functional activation and deactivation. Neurosci Biobehav Rev.

[CR14] Franklin TB, Saab BJ, Mansuy IM (2012). Neural mechanisms of stress resilience and vulnerability. Neuron.

[CR15] Goldin PR, McRae K, Ramel W, Gross JJ (2008). The neural bases of emotion regulation: reappraisal and suppression of negative emotion. Biol Psychiatry.

[CR16] Goldin PR, Moodie CA, Gross JJ (2019). Acceptance versus reappraisal: behavioral, autonomic, and neural effects. Cogn Affect Behav Neurosci.

[CR18] Gross JJ (1998). The emerging field of emotion regulation: an integrative review. Rev Gen Psychol.

[CR19] Gross JJ (2015). Emotion regulation: current status and future prospects. Psychol Inq.

[CR20] Haga SM, Kraft P, Corby EK (2009). Emotion regulation: antecedents and well-being outcomes of cognitive reappraisal and expressive suppression in cross-cultural samples. J Happiness Stud.

[CR21] Hahn RTJ, Stein EA (2006). Neuroanatomical dissociation between bottom–up and top–down processes of visuospatial selective attention. Neuroimage.

[CR22] Hammen C, Henry R, Daley SE (2000). Depression and sensitization to stressors among young women as a function of childhood adversity. J Consult Clin Psychol.

[CR23] Henckens MJ, van Wingen GA, Joëls M, Fernández G (2012). Time-dependent effects of cortisol on selective attention and emotional interference: a functional MRI study. Front Integr Neurosci.

[CR24] John OP, Naumann LP, Soto CJ, John OP, Robins RW, Pervin LA (2008). Paradigm shift to the integrative Big Five trait taxonomy: history, measurement, and conceptual issues. Handbook of personality: theory and research.

[CR25] Kim P, Evans GW, Angstadt M, Ho SS, Sripada CS, Swain JE, Liberzon I, Phan KL (2013). Effects of childhood poverty and chronic stress on emotion regulatory brain function in adulthood. Proc Natl Acad Sci USA.

[CR26] Kohn N, Eickhoff SB, Scheller M, Laird AR, Fox PT, Habel U (2014). Neural network of cognitive emotion regulation—an ALE meta-analysis and MACM analysis. Neuroimage.

[CR27] Levine S (2005). Developmental determinants of sensitivity and resistance to stress. Psychoneuroendocrinology.

[CR29] Lindstrom KM, Guyer AE, Mogg K, Bradley BP, Fox NA, Ernst M, Nelson EE, Leibenluft E, Britton JC, Monk CS, Pine DS, Bar-Haim Y (2009). Normative data on development of neural and behavioral mechanisms underlying attention orienting toward social–emotional stimuli: an exploratory study. Brain Res.

[CR30] Loeffler LAK, Satterthwaite TD, Habel U, Schneider F, Radke S, Derntl B (2019). Attention control and its emotion-specific association with cognitive emotion regulation in depression. Brain Imaging Behav.

[CR31] McRae K, Gross JJ, Weber J, Robertson ER, Sokol-Hessner P, Ray RD, Gabrieli JDE, Ochsner KN (2012). The development of emotion regulation: an fMRI study of cognitive reappraisal in children, adolescents and young adults. Soc Cogn Affect Neurosci.

[CR32] Marchewka A, Żurawski Ł, Jednoróg K, Grabowska A (2014). The Nencki Affective Picture System (NAPS): introduction to a novel, standardized, wide-range, high-quality, realistic picture database. Behav Res Methods.

[CR33] Mather M, Canli T, English T, Whitfield S, Wais P, Ochsner K, Gabrieli JDE, Carstensen LL (2004). Amygdala responses to emotionally valenced stimuli in older and younger adults. Psychol Sci.

[CR34] McEwen BS, Bowles NP, Gray JD, Hill MN, Hunter RG, Karatsoreos IN, Nasca C (2015). Mechanisms of stress in the brain. Nat Neurosci.

[CR35] McLaughlin KA, Sheridan MA (2016). Beyond cumulative risk: a dimensional approach to childhood adversity. Curr Dir Psychol Sci.

[CR36] McLaughlin KA, Peverill M, Gold AL, Alves S, Sheridan MA (2015). Child maltreatment and neural systems underlying emotion regulation. J Am Acad Child Adolesc Psychiatry.

[CR37] Mizokami Y, Terao T, Hatano K, Hoaki N, Kohno K, Araki Y, Kodama K, Makino M, Izumi T, Shimomura T, Fujiki M, Kochiyama T (2014). Difference in brain activations during appreciating paintings and photographic analogs. Front Hum Neurosci.

[CR38] Morawetz C, Bode S, Derntl B, Heekeren HR (2017). The effect of strategies, goals and stimulus material on the neural mechanisms of emotion regulation: a meta-analysis of fMRI studies. Neurosci Biobehav Rev.

[CR39] Morelli NM, Liuzzi MT, Duong JB, Kryza-Lacombe M, Chad-Friedman E, Villodas MT, Dougherty LR, Wiggins JL (2021). Reward-related neural correlates of early life stress in school-aged children. Dev Cogn Neurosci.

[CR40] Muscatell KA, Addis DR, Kensinger EA (2010). Self-involvement modulates the effective connectivity of the autobiographical memory network. Soc Cogn Affect Neurosci.

[CR41] Nederhof E, Schmidt MV (2012). Mismatch or cumulative stress: toward an integrated hypothesis of programming effects. Physiol Behav.

[CR42] Nederhof E, Ormel J, Oldehinkel AJ (2014). Mismatch or cumulative stress: the pathway to depression is conditional on attention style. Psychol Sci.

[CR44] Ochsner KN, Gross JJ (2005). The cognitive control of emotion. Trends Cogn Sci.

[CR45] Ochsner KN, Ray RD, Cooper JC, Robertson ER, Chopra S, Gabrieli JDE, Gross JJ (2004). For better or for worse: neural systems supporting the cognitive down- and up-regulation of negative emotion. Neuroimage.

[CR46] Ochsner KN, Silvers JA, Buhle JT (2012). Functional imaging studies of emotion regulation: a synthetic review and evolving model of the cognitive control of emotion. Ann N Y Acad Sci.

[CR47] Oomen CA, Soeters H, Audureau N, Vermunt L, van Hasselt FN, Manders EMM, Joëls M, Lucassen PJ, Krugers H (2010). Severe early life stress hampers spatial learning and neurogenesis, but improves hippocampal synaptic plasticity and emotional learning under high-stress conditions in adulthood. J Neurosci.

[CR48] Palgi Y, Shrira A, Ben-Ezra M, Shiovitz-Ezra S, Ayalon L (2012). Self- and other-oriented potential lifetime traumatic events as predictors of loneliness in the second half of life. Aging Ment Health.

[CR49] Paquola C, Bennett MR, Hatton SN, Hermens DF, Lagopoulos J (2017). Utility of the cumulative stress and mismatch hypotheses in understanding the neurobiological impacts of childhood abuse and recent stress in youth with emerging mental disorder. Hum Brain Mapp.

[CR50] Pechtel P, Pizzagalli DA (2011). Effects of early life stress on cognitive and affective function: an integrated review of human literature. Psychopharmacology.

[CR52] Rahe RH (1975). Epidemiological studies of life change and illness. Int J Psychiat Med.

[CR54] Santarelli S, Lesuis SL, Wang XD, Wagner KV, Hartmann J, Labermaier C, Scharf SH, Müller MB, Holsboer F, Schmidt MV (2014). Evidence supporting the match/mismatch hypothesis of psychiatric disorders. Eur Neuropsychopharmacol.

[CR55] Santos A, Mier D, Kirsch P, Meyer-Lindenberg A (2011). Evidence for a general face salience signal in human amygdala. Neuroimage.

[CR56] Sawyer SM, Azzopardi PS, Wickremarathne D, Patton GC (2018). The age of adolescence. Lancet Child Adolesc Health.

[CR57] Schwabe L, Wolf OT (2013). Stress and multiple memory systems: from ‘thinking’to ‘doing’. Trends Cogn Sci.

[CR58] Schweizer S, Walsh ND, Stretton J, Dunn VJ, Goodyer IM, Dalgleish T (2016). Enhanced emotion regulation capacity and its neural substrates in those exposed to moderate childhood adversity. Soc Cogn Affect Neurosci.

[CR59] Sobolewski A, Strelau J, Zawadzki B (1999). Kwestionariusz Zmian Życiowych (KZŻ). Przegląd Psychologiczny.

[CR60] Sokołowski A, Dragan WŁ (2017). New empirical evidence on the validity and the reliability of the early life stress questionnaire in a polish sample. Front Psychol.

[CR61] Sokołowski A, Folkierska-Żukowska M, Jednoróg K, Moodie CA, Dragan WŁ (2020). The relationship between early and recent life stress and emotional expression processing: a functional connectivity study. Cogn Affect Behav Neurosci.

[CR63] Szabo YZ, Fernandez-Botran R, Newton TL (2019). Cumulative trauma, emotion reactivity and salivary cytokine levels following acute stress in healthy women. Anxiety Stress Copin.

[CR64] Takishima-Lacasa JY, Higa-McMillan CK, Ebesutani C, Smith RL, Chorpita BF (2014). Self-consciousness and social anxiety in youth: the Revised Self-Consciousness Scales for Children. Psychol Assess.

[CR65] Todd RM, Cunningham WA, Anderson AK, Thompson E (2012). Affect-biased attention as emotion regulation. Trends Cogn Sci.

[CR66] Uddin LQ, Yeo BT, Spreng RN (2019). Towards a universal taxonomy of macro-scale functional human brain networks. Brain Topogr.

[CR67] van Marle HJF, Hermans EJ, Qin S, Fernández G (2009). From specificity to sensitivity: how acute stress affects amygdala processing of biologically salient stimuli. Biol Psychiatry.

[CR68] Vrtička P, Sander D, Vuilleumier P (2011). Effects of emotion regulation strategy on brain responses to the valence and social content of visual scenes. Neuropsychologia.

[CR69] Vrticka P, Simioni S, Fornari E, Schluep M, Vuilleumier P, Sander D (2013). Neural substrates of social emotion regulation: a fMRI study on imitation and expressive suppression to dynamic facial signals. Front Psychol.

[CR71] Wadden KP, Snow NJ, Sande P, Slawson S, Waller T, Boyd LA (2018). Yoga practitioners uniquely activate the superior parietal lobule and supramarginal gyrus during emotion regulation. Front Integr Neurosci.

[CR72] Wager TD, Davidson ML, Hughes BL, Lindquist MA, Ochsner KN (2008). Prefrontal-subcortical pathways mediating successful emotion regulation. Neuron.

[CR73] Wu G, Feder A, Cohen H, Kim JJ, Calderon S, Charney DS, Mathé AA (2013). Understanding resilience. Front Behav Neurosci.

[CR74] Yeo BT, Krienen FM, Sepulcre J, Sabuncu MR, Lashkari D, Hollinshead M, Roffman JL, Smoller JW, Zöllei L, Polimeni JR, Fischl B, Liu H, Buckner RL (2011). The organization of the human cerebral cortex estimated by intrinsic functional connectivity. J Neurophysiol.

[CR75] Zhang W, He X, Lai S, Wan J, Lai S, Zhao X, Li D (2017). Neural substrates of embodied natural beauty and social endowed beauty: an fMRI study. Sci Rep.

