# Supplementary material for: It is Not (Always) the Mismatch That Beats You—On the Relationship Between Interaction of Early and Recent Life Stress and Emotion Regulation, an fMRI Study
Source: Brain Topogr. 2021 Nov 14;35(2):219–31. doi: 10.1007/s10548-021-00880-y (PMC8860803; doi:10.1007/s10548-021-00880-y)
Supplement: Supplementary file 1 — Supplementary file1 (PDF 1491 kb) [file 10548_2021_880_MOESM1_ESM.pdf]

Supplementary Information for the article:

It is not (always) the mismatch that beats you - on the relationship between interaction of early and recent life stress and emotion regulation, an fMRI study

Andrzej Sokołowski[1], Monika Folkierska-Żukowska[2], Katarzyna Jednoróg[3], Marek Wypych[4], Wojciech Ł. Dragan[2*]

[1]Department of Neurology, Memory and Aging Center, UCSF Weill Institute for Neurosciences, University of California, San Francisco, San Francisco, CA, USA

[2]The Interdisciplinary Centre for Behavioral Genetics Research, University of Warsaw, Warsaw, Poland

[3]Laboratory of Language Neurobiology, Nencki Institute of Experimental Biology of Polish Academy of Sciences, Warsaw, Poland

[4]Laboratory of Brain Imaging, Nencki Institute of Experimental Biology of Polish Academy of Sciences, Warsaw, Poland

*Corresponding Author:
Wojciech Łukasz Dragan
Faculty of Psychology
University of Warsaw,
Warsaw, Poland
Postal address: Stawki 5/7, 00-183 Warszawa, Poland
Email: wdragan@psych.uw.edu.pl

Correspondence may also be addressed to Andrzej Sokołowski
Email: andrzej.sokolowski@ucsf.edu

Supplementary Information

Community sample

Participants were selected from a larger community sample (N = 503; 250 men, 253 women). They were recruited from Warsaw, Poland and surrounding area. Age ranged 18 – 25 (M = 21.41; SD = 1.88). Years of education ranged 1 – 22 (M = 13.21; SD 2.71). Early Life Stress Questionnaire scores ranged 0 – 13 (M = 2.97; SD = 2.93). Recent Life Changes Questionnaire scores ranged 0 – 42 (M = 11.10; SD = 7.28).

Stimuli

The emotional stimuli were chosen from a standardized database based on arousal and valence values. Inclusion criteria were the presence of a social situation, people, or faces—i.e., a social stimulus that can be reappraised. Valence values ranged from 0 to 9, with low values corresponding to negative valence, intermediate values corresponding to neutral valence, and high values corresponding to positive valence. Arousal values ranged from 0 to 9, with higher values indicating higher arousal. The valence scores for negative photos were between 2.13 and 3.94; neutral pictures between 4.84 and 5.71; and positive pictures between 6.27 and 8.22. The arousal scores for neutral images were between 3.69 and 5.07; negative pictures between 5.42 and 6.56; and positive pictures between 4.50 and 5.82. Descriptive statistics are presented in Table S1. Three groups of pictures differed with regard to valence. Unfortunately, due to the insufficient number of pictures in the database meeting the inclusion criteria and the fact that the pictures with a negative valence were usually rated as more arousing than pictures with positive valence, positive and negative stimuli differed with regard to arousal. Pictures with negative valence were significantly more arousing than those with positive valence (p <.001).

Table S1
Emotional stimuli used in the reappraisal task.

	Valence		
	Neutral M (SD)	Positive M (SD)	Negative M (SD)
Valence	5.28 (.26)	7.24 (.43)	3.22 (.46)
Arousal	4.68 (.34)	5.02 (.35)	6.13 (.29)

Cognitive reappraisal task

There were 7 task conditions: 2 regulation goals (increase, decrease) x 2 stimuli valence (negative, positive), as well as look control conditions with negative, positive, and neutral stimuli. A block design was used. The task consisted of 4 blocks of each condition (28 blocks in total). The order of the blocks was pseudorandomized, with the restriction that no more than 2 blocks of the same condition could appear after one another. The task design is shown in Supplementary Fig.S1. Each block started with a 5s instruction (decrease, increase, or look), followed by 4 consecutive pictures of the same emotional valence (positive, negative, or neutral). Images were presented for 5s in a fully randomized fashion for all conditions. Visual symbols were displayed under each picture to cue participants to the current regulation goal: a downward arrow (decrease), an upward arrow (increase), or a dash (look). Participants rated the strength of their current emotions after the regulation phase. Affect rating was displayed on the screen until the response was given, followed by a fixation cross for a total duration of 15s. After the rating, a fixation cross was displayed until the next block. Affect rating was registered as a behavioural measure by pressing and holding a button on a pad held in the right hand until an affect rating 'thermometer' reflected the subject's affect. The visual affect scale was converted to a continuous scale which scores ranged from 0 (low affect) to 100 (high affect).

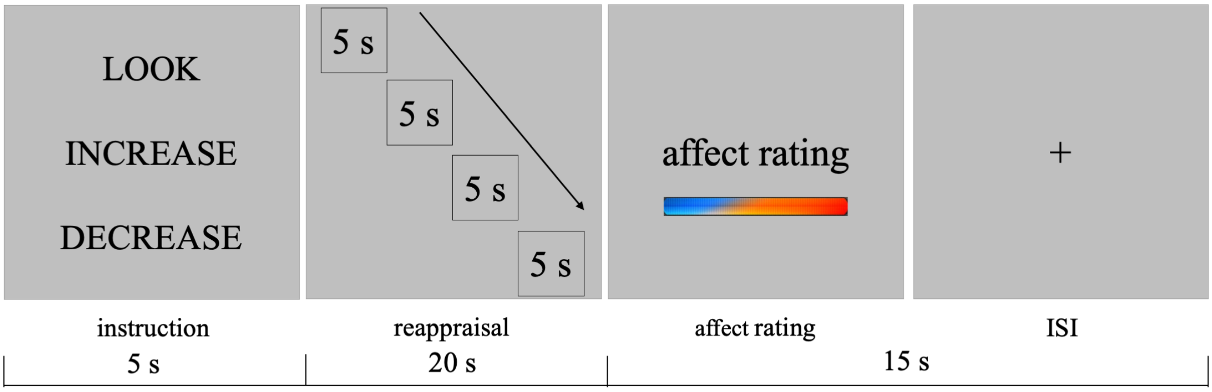

Fig.S1. Reappraisal task design. Each block started with an instruction followed by 4 pictures of the same valence (positive, negative, or neutral). Affect rating was displayed after the regulation phase followed by a fixation cross until next experimental block.

Whole-brain activation

Main effect of task (decrease + increase > look contrast)

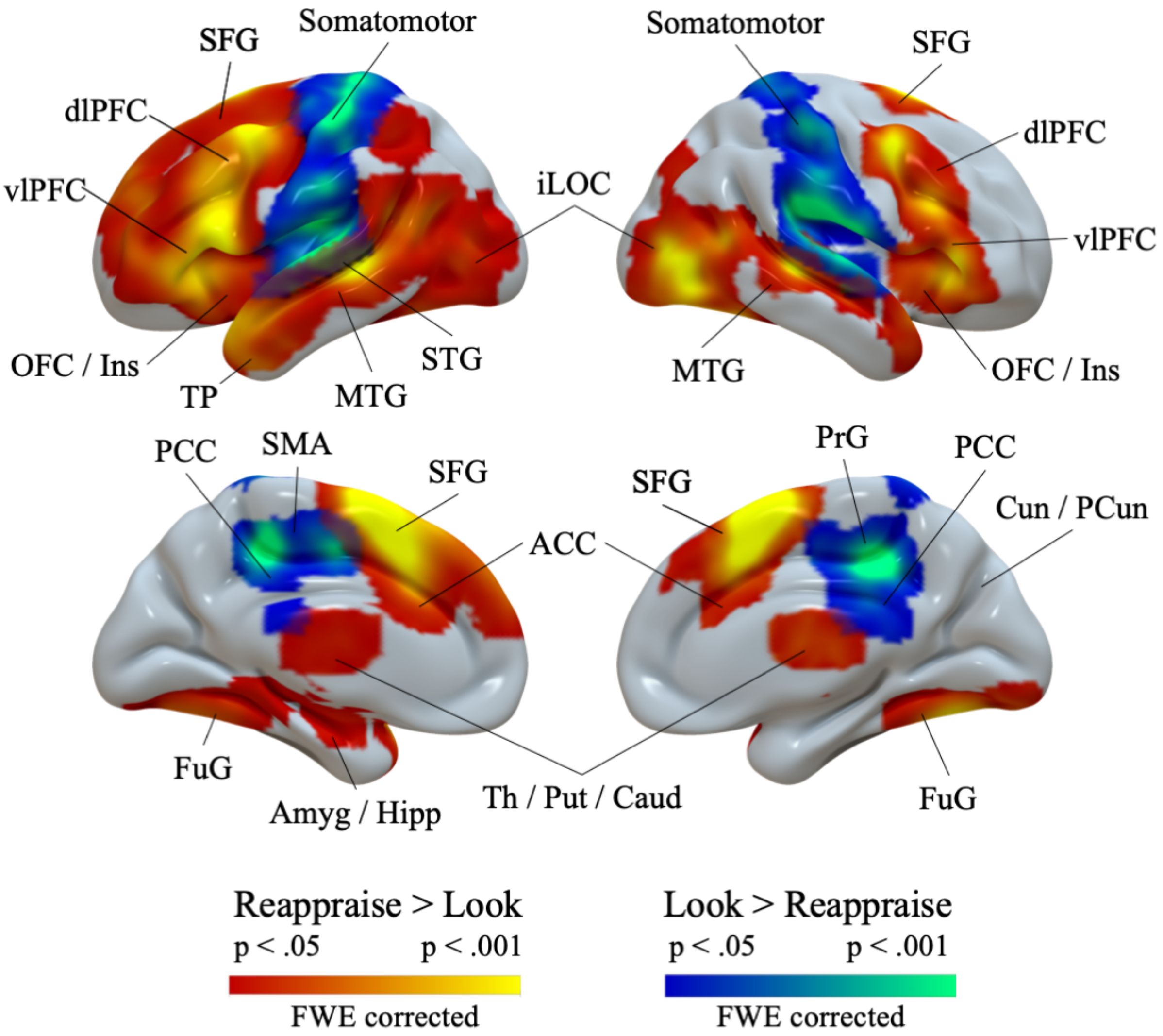

Fig.S2. Reappraisal-related brain activation across all participants (decrease + increase > look contrast). Warm colors indicate stronger activation during reappraisal; cool colors indicate stronger activation during control condition. Abbreviations: ACC, anterior cingulate cortex; Amyg, amygdala; Caud, caudate; Cun, cuneus; dlPFC, dorsolateral prefrontal cortex; FuG, fusiform gyrus; Hipp, hippocampus; iLOC, inferior lateral occipital cortex; Ins, insula; MTG, middle temporal gyrus; OFC, orbitofrontal cortex; PCC, posterior cingulate gyrus; PCun, precuneus; PrG, precentral gyrus; Put, putamen; SFG, superior frontal gyrus; SMA, supplementary motor area; STG, superior temporal gyrus; Th, thalamus; TP, temporal pole; vlPFC, ventrolateral prefrontal cortex.

Table S2

Reappraisal-related brain activation for the main effect of task (decrease + increase > look contrast).

Brain Region(s)		Peak X (MNI)	Peak Y (MNI)	Peak Z (MNI)	T	Cluster Size (voxels)
Activation						
SMA / SFG	L	-4	6	64	11.15	32623
SMA	L	-8	10	56	10.89	lm
vlPFC	L	-50	18	0	10.00	lm
dlPFC	L	-36	4	56	9.41	lm
Caud	R	18	2	18	6.86	1078
GP	R	18	0	0	4.90	lm
Put	R	24	8	4	4.44	lm
vlPFC	R	52	24	0	6.67	3958
dlPFC	R	46	6	48	6.11	lm
Insula	R	46	14	0	5.70	lm
MTG	R	48	-36	0	5.89	570
STG	R	50	-14	-12	4.18	lm
SPL	L	-38	-54	50	4.48	332
**Deactivation**						
PoG	L	-44	-32	64	6.64	3234
PoG	L	-24	-34	76	6.38	lm
PoG	L	-50	-28	58	6.19	lm
CO	L	-60	-20	10	6.06	2592
CO	L	-40	-20	20	5.12	lm
Insula	L	-38	-4	14	5.09	lm

CO / PT	R	62	-14	10	5.66	2889
SMG	R	50	-32	56	5.37	lm
PO	R	50	-30	28	5.35	lm

Abbreviations: Caud, caudate; CO, central operculum; Cun, cuneus; dlPFC, dorsolateral prefrontal cortex; GP, globus pallidus; L, left hemisphere; MTG, middle temporal gyrus; PCun, precuneus; PO, parietal operculum; PoG, postcentral gyrus; PT, planum temporale; Put, putamen; R, right hemisphere; SMA, supplementary motor area, SMG, supramarginal gyrus; SPL, superior parietal lobule; STG, superior temporal gyrus; vlPFC, ventrolateral prefrontal cortex.

Effects of early and recent life stress

In line with the analyses regarding cumulative and mismatch stress, 2x2 flexible factorial model was used with stimuli valence (negative, positive) and regulation goals (increase, decrease) and their interactions with the early and recent life stress. To compare the effects of early and recent life stress the two stress regressors were directly compared for each of four reappraisal task conditions. Family-wise error (FWE) correction was used to control for multiple comparisons in whole-brain analysis ($p < .001$ height-threshold; FWE < .05 extent-threshold).

Interaction with stimuli valence and regulation goal

There was a significant interaction between early life stress and stimuli valance (Table S3). There were no significant interactions with regulation goals or recent life stress.

Table S3

Interaction between early life stress and stimuli valence in the whole-brain activation during reappraisal

Contrast and Brain Region(s)	Cluster Size (voxels)	p-value for cluster (FWE)	F	Peak X (MNI)	Peak Y (MNI)	Peak Z (MNI)
Early life stress x valence						
Cerebellum / Thalamus / Putamen / Insula	5069	< .001	32.82	-2	-30	-14
Cuneus / sLOC	964	< .001	30.72	-20	-80	20
SPL	1105	< .001	29.95	18	-54	48
MTG	373	.02	25.74	54	-52	6
Caudate	294	.045	25.49	-14	22	16

sLOC / SPL	522	.005	25.12	-16	-60	46
Precentral / MFG	1239	< .001	23.15	42	-2	44
Pallidum / Thalamus	435	.011	21.06	22	-10	-4
sLOC / Cuneus	818	< .001	20.5	24	-70	22
Hippocampus	291	.047	18.76	34	-34	-8

MFG – middle frontal gyrus; MTG – middle temporal gyrus; sLOC – superior lateral occipital cortex; SPL – superior parietal lobule.

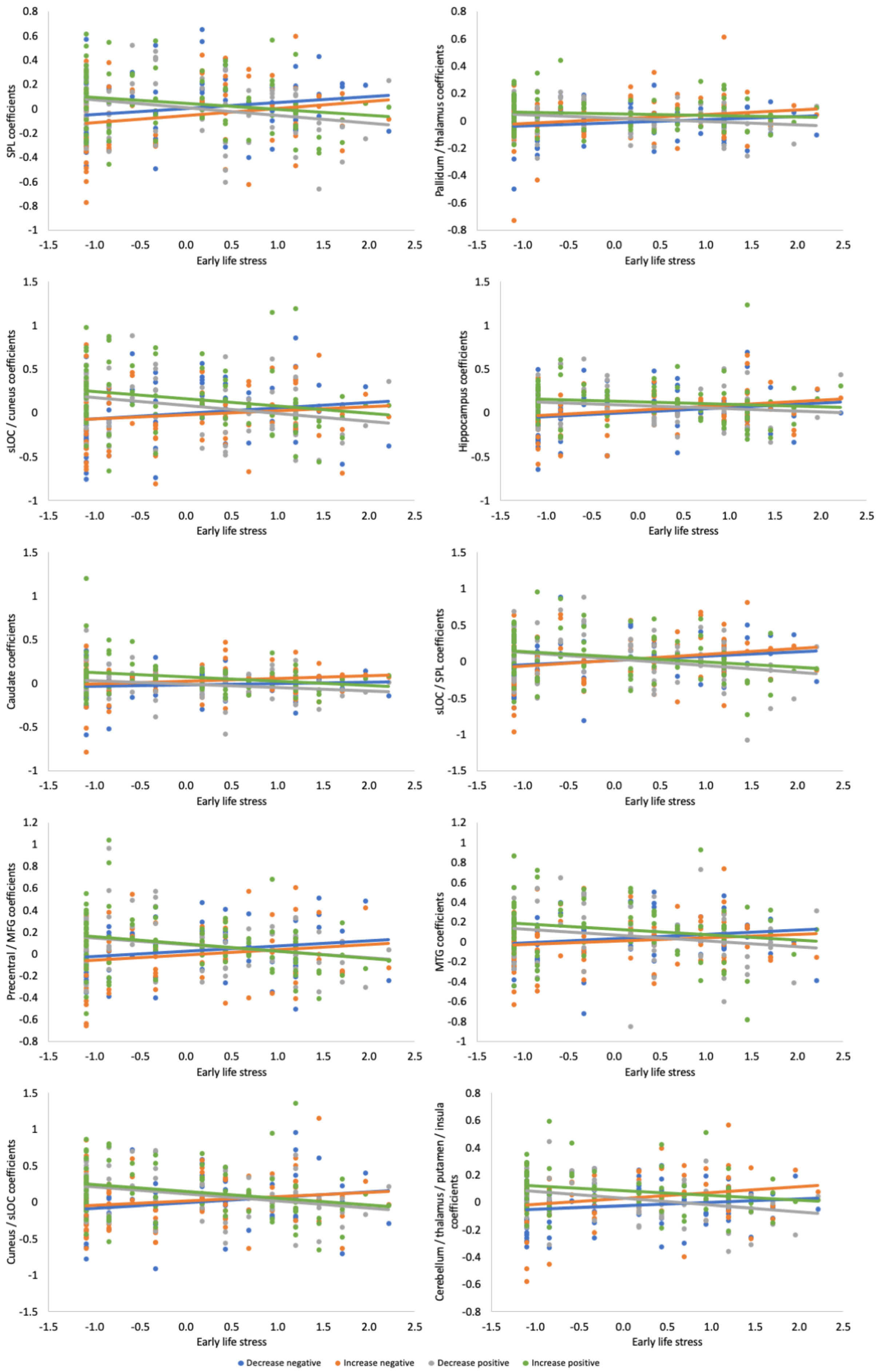
SPL coefficients
Early life stress
Pallidum / thalamus coefficients
Early life stress
sLOC / cuneus coefficients
Early life stress
Hippocampus coefficients
Early life stress
Caudate coefficients
Early life stress
sLOC / SPL coefficients
Early life stress
Precentral / MFG coefficients
Early life stress
MTG coefficients
Early life stress
Cuneus / sLOC coefficients
Early life stress
Cerebellum / thalamus / putamen / insula coefficients
Early life stress
Decrease negative
Increase negative
Decrease positive
Increase positive

Fig.S3. Interaction effect between early life stress and stimuli valence on brain activation. Regression slopes for all task conditions are presented. Standardized early life stress scores are presented on x axis.

Effects of early and recent life stress on whole-brain activation in four task conditions

There was a difference between the effects of early and recent life stress on brain activation during increasing negative emotions (Table S4). There were no significant results for other task conditions.

Table S4

Reappraisal-related brain activation for the increase negative > view negative contrast

Contrast and Brain Region(s)	Cluster Size (voxels)	p-value for cluster (FWE)	t	Peak X (MNI)	Peak Y (MNI)	Peak Z (MNI)
Positive correlation with early life stress						
SFG / Precentral	621	.004	4.36	-14	-10	58
Thalamus	506	.009	4.25	-18	-22	-2
Early stress > Recent stress						
Putamen / Thalamus / Hippocampus	497	.01	4.28	-24	-18	-2
SMG / Postcentral	316	.049	4.19	66	-16	36
Precentral / SFG	566	.005	4.16	-14	-10	56

SFG – superior frontal gyrus; SMG – supramarginal gyrus.
